# Are we ready for artificial intelligence health monitoring in elder care?

**DOI:** 10.1186/s12877-020-01764-9

**Published:** 2020-09-21

**Authors:** Anita Ho

**Affiliations:** 1 Centre for Applied Ethics, University of British Columbia, 227 – 6356 Agricultural Road, Vancouver, BC V6T 1Z2 Canada; 2 Bioethics Program, University of California San Francisco, Vancouver, Canada; 3 Centre for Health Evaluation & Outcome Sciences, Vancouver, Canada

**Keywords:** Artificial intelligence, Machine learning, Ethics, Aging in place, Independent living, Health monitoring

## Abstract

**Background:**

The world is experiencing a dramatic increase in the aging population, challenging the sustainability of traditional care models that have relied on in-person monitoring. This debate article discusses whether artificial intelligence health monitoring may be suitable enhancement or replacement for elder care.

**Main text:**

Internationally, as life expectancy continues to rise, many countries are facing a severe shortage of direct care workers. The health workforce is aging, and replacement remains a challenge. Artificial intelligence health monitoring technologies may play a novel and significant role in filling the human resource gaps in caring for older adults by complementing current care provision, reducing the burden on family caregivers, and improving the quality of care. Nonetheless, opportunities brought on by these emerging technologies raise ethical questions that must be addressed to ensure that these automated systems can truly enhance care and health outcomes for older adults. This debate article explores some ethical dimensions of using automated health monitoring technologies. It argues that, in order for these health monitoring technologies to fulfill the wishes of older adults to age in place and also to empower them and improve their quality of life, we need deep knowledge of how stakeholders may balance their considerations of relational care, safety, and privacy.

**Conclusion:**

It is only when we design artificial intelligence health monitoring technologies with intersecting clinical and ethical factors in mind that the resulting systems will enhance productive relational care, facilitate independent living, promote older adults’ health outcomes, and minimize waste.

## Background

The world is experiencing a dramatic increase in the aging population, challenging the sustainability of traditional care models that have relied on in-person monitoring. The global population age 65 and over is projected to double from 8.5% of the world’s population (617 million) to 17% by 2050 (1.6 billion) [1]. Longer life expectancy often means living with impairments and chronic conditions that may affect people’s ability to perform daily activities or function independently [2]. Compounding the pressure of an aging population that requires higher levels of personal attention, assistance, and care, many countries are facing a severe shortage of direct care workers such as home health aides. The health workforce is aging, and replacement remains a challenge [3]. Informal caregivers across the world, who are predominantly women, often have to juggle other personal, familial, and professional responsibilities in addition to providing constant direct care and monitoring for their elderly loved ones [4, 5]. As a result of the changing nature of family relationships, declining family size, women’s growing participation in the workforce, and migration patterns, the number of potential family caregivers per older adult is also expected to continue to drop sharply [6, 7]. Many older adults value independent living or aging in place, i.e., they prefer to live in their own home with appropriate support rather than move into institutional care [8, 9], which is also in short supply and can be beyond the means for many older adults [10]. The current COVID-19 pandemic, which has disproportionately burdened older adults, especially those in long-term care facilities, reinforces calls for further strategies to help people to age in place to the greatest extent possible and/or receive health monitoring that requires minimal in-person contact.

Remote monitoring technologies, such as those that use video cameras to observe people’s activities at home, may provide some assistance to support older adults to live independently. Nonetheless, these technologies continue to rely on human operators or family caregivers to be watching video feeds in real-time and responding accordingly based on their judgments. Thus, they are labor intensive and can be prone to human distractions and errors [11]. As we face growing human resource challenges, can new automated and continuous technologies such as artificial intelligence (AI) health monitoring enhance older adults’ ability to live safely in their desired settings?

## Main text

### What can AI health monitoring do for older adults?

Many commercial devices and systems, including those that are built into smartphones that are readily accessible to mass consumers, already collect wellness data (e.g., physical activities, dietary information) that can provide a snapshot of an older adult’s general lifestyle. These recreational products are usually not subjected to the same regulatory control as medical devices or applications that specifically collect health data (e.g., blood pressure, ECG reports). AI-powered health monitoring technologies build upon these capabilities but go beyond collecting and tracking various indicators. Endowed with processes that mimic human intelligence, such as recognizing, learning, reasoning, adapting, predicting, and deciding, AI health monitoring may play a novel and significant role in caring for older adults by complementing current care provision, reducing the burden on family caregivers, and improving the quality of care [12, 13].

Machine learning is a subset of AI that uses statistical techniques to empower computer programs to make predictions and decisions on their own based on past data, allowing the programs to perform tasks progressively better through iterative experiences. These optimization algorithms can continually collect and analyze a vast amount of data from longitudinal observation, identify and categorize patterns, and use predictive analytics to assess risk level and make behavioral or care recommendations. For example, AI-enabled blood pressure or electrocardiogram monitors may help to predict various health concerns (e.g., hypertension, atrial fibrillation). More sophisticated AI home-health monitoring system such as computer vision analytics can classify activities such as standing or walking, and then iteratively learn what is expected movement or activities for a particular older adult in a specific setting [14–19], such as the ease and the amount of time a person spends getting out of bed. Sensors that are installed in various locations in a person’s home and can track the older adult’s total daily activity, time out of home, walking speed, and location at the home, etc., may also help to register the individual’s types, sequences, and durations of activities [20]. They can identify unusual movements and activities that may suggest cognitive and functional decline. For example, AI monitoring programs that continually analyze input data may be able to detect that an older person is taking gradually longer time to gain balance while trying to stand up or regaining balance; something that a human may miss. Upon predicting health decline based on the collected data, the automated analytic system can then decide to intervene based on pre-set risk threshold by sending forewarning messages or even behavioral suggestions to the older person (and/or their caregiver) [21]. In providing not only descriptive real-time information but also automated alerts to facilitate timely and safe care, these technologies can potentially prevent acute deteriorations or serious injuries, thereby delaying or avoiding the use of costly institutional care [22, 23]. A few studies indicate that community-dwelling older adults and their family caregivers experience a greater sense of safety using automated monitoring systems at home [24–26], suggesting that these technologies may be acceptable alternatives or enhancement to in-person monitoring to support independent living [27].

At the health system level, clinically meaningful and actionable AI monitoring data regarding older adults’ risk levels for adverse events can inform medical decision making and transform healthcare delivery. They can help healthcare providers to triage cases to ensure that the right patients have timely access to appropriate care [28], which can in turn enhance clinical workflow and productivity. These AI technologies may optimize the limited human resources and equalize opportunities for older adults to age safely in their desired settings, thereby reducing access barriers and caregiver burden [29]. If integrated with older adults’ electronic health records, the monitoring technologies can further facilitate therapeutic interactions, and may be particularly helpful to support elderly patients who have difficulty articulating their symptoms or needs, including those with cognitive or memory decline. Using continuous data may also enable a better understanding of the dynamic nature of an older adult’s disease progression or functioning, since health assessment is not based solely on a set of markers at one time point but rather on how the relevant markers may vary continuously over time under identifiable circumstances tracked by the monitoring devices [30]. With the information already synthesized by the algorithms for review, the output from these monitoring systems may strengthen virtual support, preventive care, and in-person consultation. For example, during clinic visits, doctors who have access to synthesized outputs from these automated systems can shift from dispiriting documentation and data-entry tasks to the relational, therapeutic, and patient-focused activity [11]. They can review the previously collected and analyzed data and ask follow-up questions that can augment their ability to diagnose and/or manage the patient’s situation.

### Intersecting ethical considerations of using AI health monitoring

Nonetheless, opportunities brought on by emerging AI health monitoring technologies raise ethical questions that must be addressed to ensure that these automated systems can truly enhance care and health outcomes for older adults [31]. While AI technologies could theoretically facilitate early detection of declines and enable timely intervention, a systematic review studying sensor monitoring as a method to measure and support daily functioning for older adults living independently at home shows that there is currently only limited evidence of such effectiveness due to a lack of high methodological quality in relevant studies, and that most of these technologies are still in early stages of development or refinement [32]. Moreover, while medical devices have to follow basic regulatory requirements, particularly around clinical safety and data privacy, different countries have varying approaches, especially when certain commercial monitoring devices do not strictly fall under the category of medical devices [33]. Moreover, a significant portion of current AI health technologies is designed without explicit ethical considerations regarding the impact of these technologies on other stakeholders (e.g., caregivers) or the users’ relationship with others (e.g., family members, healthcare providers) [13], which may limit the responsive and ethical translational potential of these technologies in people’s homes.

As AI health monitoring developers continue to improve their technologies, this is the opportune time to incorporate ethical considerations into the design and implementation planning of these emerging technologies. We need to keep in mind that even if all stakeholders have the same goal regarding the use of AI health monitoring – i.e., to improve older adults’ ability to age in place safely – older adults, family caregivers, healthcare professionals, and insurance companies may have different perspectives regarding how this goal should be achieved, or how competing priorities should be balanced in achieving this goal. For example, older adults’ risk perceptions and risk tolerance in the context of independent living may differ from those of their family and professional caregivers [34]. In one study with Meals on Wheels clients and their adult children regarding their perceptions of in-home health monitoring technologies, researchers found that the children preferred these technologies more than their elderly parents [35]. As health care funding decisions made by insurance and other health coverage payment schemes are increasingly tied to the adoption of electronic and digital practices, these entities may also prioritize autonomous rather than in-person monitoring regardless of older adults’ preferences. Potentially divergent perspectives among different stakeholders may raise ethical questions of how we can develop and utilize AI monitoring technologies that will truly promote older adults’ autonomy and well-being, assist family and professional caregivers’ work, and prevent inadvertent infringement of older adults’ self-determination.

Exploration of older adults’ goals and priorities in using health monitoring platforms may help to develop and implement technologies that can truly promote older adults’ autonomy. One study shows that older adults are interested in co-designing these technologies and controlling their data [36]. Other studies also show that older adults wish to have control over what information AI systems may share with family and caregivers [37], and when they should be monitored to protect privacy [38]. Nonetheless, many existing monitoring systems are relatively fixed and difficult to customize according to the users’ preferences [39]. Moreover, prescriptive outputs from AI predictive analytics such as behavioral change recommendations may counter an older adult’s desire or their own risk assessment (e.g., getting out of bed without assistance). While disagreement with professional recommendation is not new, as people tend to consider algorithms as being more objective than humans, recommendations from AI health monitoring may inadvertently intensify power and control [40]. We can imagine that care providers may use algorithmic suggestions to reinforce their own recommendations for action, or that older adults may face additional scrutiny from their family or professional caregivers if they reject the safety recommendations put forth by the algorithm, potentially restricting rather than expanding the activities of these older adults [41]. If monitoring technologies may impose “objective” recommendations regardless of older adults’ own priorities and concerns, to retain or regain some levels of control, users may simply abandon the technology or try to manipulate the data (e.g., find various means to trick the system) [42].

Health monitoring and care management, even with the assistance of AI technologies, are done in and through relationships between dependent people and those who care for them [43]. It occurs within a multidimensional and interdependent context with reference to social roles and power structure, shaped by interpersonal dynamics, the contemporary medical culture, institutional and funding frameworks, and the technological imperatives. The intersectionality of older adults’ functional and cognitive abilities, cultural backgrounds, health/digital literacy, and desired relationship with family/professional caregivers may influence their attitudes towards AI health monitoring and information sharing preferences. Efforts to promote older adults’ autonomy and cost-effective health monitoring thus require a reflective understanding of the multi-level considerations framing the ways older adults and prospective caregivers receive, provide, or utilize AI health monitoring.

At the individual and family levels, we need to consider how older adults may perceive the utility, acceptability, and trustworthiness of AI health monitoring technologies for everyday care [44, 45]. As AI technologies collect, store, and process data to provide predictions and/or recommendations, there are questions of how older adults and their families may weigh personal and data privacy considerations against gains in independent living, physical safety, and convenience [30]. In one study with elder residents, family members, and care staff of an independent living complex where passive health monitoring is offered, staff participants and older adults who consented to monitoring reported different boundaries and approaches to privacy [38]. It is also noteworthy that many people, particularly those living in family-centric cultures [46], highly value family care relationships, raising questions of how AI technologies that replace and yet facilitate various aspects of caregiving work may affect family dynamics. In one study assessing older adults’ experience using a remote health monitoring system that collects biometric information and addresses health topics such as nutrition, diabetes, cardiac care, and medication compliance, the longer participants used the technology, the more they perceived those important to them would want them to use it, raising questions of how we must navigate the extent of social influence on older adults’ technology adoption [47]. Another study with older adults and their family caregivers shows that health monitoring technologies may transform existing hidden care routines between family members into care work [48]. As consent and the use of monitoring technologies is often carried out in a familial care setting, there are also pertinent ethical questions of how we should balance older adults’ autonomy, privacy, and well-being with the interests and preferences of family caregivers [49]. Some individuals’ capacity to (withdraw) consent to continuous observation and longitudinal data sharing may gradually decline as their various conditions progress. Ironically, this is also when people’s risk of unobserved health deterioration will likely increase, raising questions of how (much) to involve these older adults in deciding the utilization of these technologies, and who should hold decisional power of usage and discontinuance [26].

At the organizational and health system levels, we need to ensure that the promises of AI health technologies do not inadvertently lead to the collection and incorporation of clinically irrelevant data that may further burden older adults and their family caregivers, which can in turn also waste valuable healthcare resources [50]. As direct-to-consumer technologies have increasingly blurred recreational and health information, and that insurance companies and health systems are increasingly preferring electronic practices and digital data for care delivery, older adults, family caregivers, healthcare professionals, and funding decision makers may need guidance on distinguishing high-value and clinically meaningful data that can truly guide or affect care decisions from “biomarkup” that may indicate risks for diseases before there is reliable data showing that further investigations would likely improve health outcomes [51]. In considering the adoption of AI monitoring technologies, various organizations and health systems also need to balance human resource and cost factors in exploring the most efficient and effective ways to provide safe care for their older clients, including whether or how the utilization of AI monitoring technologies will align with various quality assurance/improvement initiatives. Given that effective operationalization of AI monitoring would require upfront costs, significant training, and behavior change interventions throughout the organizational and system levels, it is also important to consider how stakeholders at different levels perceive such investments and disruptive changes.

At the societal level, existential, operational, and ethical questions abound. As AI monitoring technologies and new data collection/sharing practices blur the line between private and health information, utilization of these technologies may affect people’s views about health, wellness, illness, and what it means to be a patient [52]. We need to consider how enthusiasm and investment in AI monitoring technologies may juxtapose with governments’ commitments in healthcare workforce training and planning. Keeping in mind broader justice concerns, there are also questions of how we can ensure that automated and remote observation technologies can be a platform to provide older adults with more appropriate contact with the healthcare system rather than exacerbate the digital divide or social isolation [30, 53, 54]. The issue of potential isolation is of both clinical and ethical concerns because it has been associated with self-report of poor health and increased risk for early mortality [55].

### Promoting care relationships and health outcomes with AI technologies

Older adults desire to age in place *and* in relationships. Thus, in addition to clinical health outcomes, we also need to consider how AI health monitoring technologies may affect older adults’ emotional, psychosocial, and relational dimensions [13]. The ethical value of health technologies, including AI health monitoring technologies, lies partly in their potential to enhance familial and professional care relationships that can in turn fulfill older adults’ desire to age in place with appropriate support [56]. The concept of independent living espouses the idea that all people, including older adults who may have limited or declining functional capacities, should have the same right to live free and independent lives in the community and to make decisions for themselves without paternalistic prejudice by others [57]. Independent living or autonomy in this context is relational. It is about people’s ability to participate, cohabitate, and interact with family and others in ways they see fit rather than a rejection of personal assistance by others or a desire to live in isolation.

Multidisciplinary research exploring the perspectives of different stakeholders (e.g., older adults, family caregivers, healthcare providers, AI engineers, marketers, and ethicists) in developing and utilizing these technologies may provide an important feedback loop to ensure responsible and responsive technology development and implementation. In the process of identifying ethical ways to harness the potential benefits of innovative AI health monitoring technologies, empirical exploratory research regarding how the concepts of relationality, autonomy, and independent living are realized or compromised in the AI health monitoring practice can inform practical solutions to potential ethical problems of utilizing such technologies [58]. Knowledge about how older adults compare human to AI monitoring, what types of privacy and care relationships are most important in enhancing health outcomes [37], and what information professionals consider to be most valuable to promote care management and workflow, may help AI health developers to focus on the relevant privacy and clinical features that can augment caregivers’ ability to provide tailored monitoring and care management plans. For example, knowledge regarding what general health and activity information older adults would like to have collected (and not collected), whether or how they might wish to interact with the technologies and the data as part of self-management, and what information they would like to share (and not share) with family members, professionals, and external commercial entities can clarify how older adults may balance considerations of privacy, confidentiality, convenience, and independence related to AI home-based health monitoring. Partnering with these stakeholders in designing the implementation of health monitoring technologies may help to yield new insight on how older adults may use these automated platforms to facilitate aging in place, what types of tradeoffs they might consider (e.g., privacy vs safety benefits), and how these technologies may affect therapeutic encounters and relationships [59]. And since a major goal of developing AI health monitoring technologies is to provide continuous and actionable information, research regarding what family and professional caregivers consider to be high-value information that can facilitate effective beside care, long-term care management, and therapeutic alliance to improve health outcomes would be pertinent.

## Conclusion

Automated monitoring systems that can provide a comprehensive picture of the older adults’ overall activity patterns and the environmental contexts within which various symptoms manifest can be valuable tools for healthy aging. These technologies, if developed ethically, can be cognitive assistants for older adults, family caregivers, and healthcare professionals [12]. They can complement current care provision, reduce the burden on unpaid caregivers, and improve the quality of care [13]. Such improvement may help to delay or obviate the need for institutional care for a rapidly expanding elderly population, thereby promoting older adults’ autonomy and simultaneously alleviating the burden on public finances. Nonetheless, in order for AI health monitoring technologies to fulfill the wishes of older adults to age in place and also to empower them and improve their quality of life, we need deep knowledge of how different stakeholders may balance their divergent considerations of relational care, safety, and privacy. It is only when we design these technologies with intersecting clinical and ethical factors in mind that the resulting systems will enhance productive relational care, facilitate independent living, promote health outcomes, and minimize waste.

## Data Availability

Not applicable.

## Abbreviation

AI: Artificial intelligence
